# Deep Learning-Assisted Repurposing of Plant Compounds for Treating Vascular Calcification: An In Silico Study with Experimental Validation

**DOI:** 10.1155/2022/4378413

**Published:** 2022-01-05

**Authors:** Chia-Ter Chao, You-Tien Tsai, Wen-Ting Lee, Hsiang-Yuan Yeh, Chih-Kang Chiang

**Affiliations:** ^1^Nephrology Division, Department of Internal Medicine, National Taiwan University Hospital, Taipei, Taiwan; ^2^Graduate Institute of Toxicology, National Taiwan University College of Medicine, Taipei, Taiwan; ^3^Nephrology Division, Department of Internal Medicine, National Taiwan University College of Medicine, Taipei, Taiwan; ^4^School of Big Data Management, Soochow University, Taipei, Taiwan

## Abstract

**Background:**

Vascular calcification (VC) constitutes subclinical vascular burden and increases cardiovascular mortality. Effective therapeutics for VC remains to be procured. We aimed to use a deep learning-based strategy to screen and uncover plant compounds that potentially can be repurposed for managing VC.

**Methods:**

We integrated drugome, interactome, and diseasome information from Comparative Toxicogenomic Database (CTD), DrugBank, PubChem, Gene Ontology (GO), and BioGrid to analyze drug-disease associations. A deep representation learning was done using a high-level description of the local network architecture and features of the entities, followed by learning the global embeddings of nodes derived from a heterogeneous network using the graph neural network architecture and a random forest classifier established for prediction. Predicted results were tested in an *in vitro* VC model for validity based on the probability scores.

**Results:**

We collected 6,790 compounds with available Simplified Molecular-Input Line-Entry System (SMILES) data, 11,958 GO terms, 7,238 diseases, and 25,482 proteins, followed by local embedding vectors using an end-to-end transformer network and a node2vec algorithm and global embedding vectors learned from heterogeneous network via the graph neural network. Our algorithm conferred a good distinction between potential compounds, presenting as higher prediction scores for the compound categories with a higher potential but lower scores for other categories. Probability score-dependent selection revealed that antioxidants such as sulforaphane and daidzein were potentially effective compounds against VC, while catechin had low probability. All three compounds were validated *in vitro*.

**Conclusions:**

Our findings exemplify the utility of deep learning in identifying promising VC-treating plant compounds. Our model can be a quick and comprehensive computational screening tool to assist in the early drug discovery process.

## 1. Introduction

The ectopic deposition of calcium apatite crystals in vascular walls, or vascular calcification (VC), emerges as a silent killer for populations with chronic kidney disease (CKD), diabetes mellitus (DM), and those of advanced age [1]. VC can be detected in as early as the fourth decade of life and becomes nearly universal in those older than 65 years, progressing from the abdominal aorta to the ascending aorta at a later time [2]. Meta-analyses showed that the prevalence of coronary or aortic VC reached as high as 65% to 70% [3, 4]. VC leads to vascular stiffening, reducing major vessel compliance and increasing cardiac afterload, culminating in myocardial hypertrophy, heart failure, and cardiovascular mortality. Patients with CKD are at a particularly high risk [5], and they frequently develop occult vascular pathologies especially VC. The perverse influences conferred by VC therefore warrants complete understandings of its pathophysiology and fuel searches for potential candidates to treat VC [6, 7].

Processes responsible for the development and aggravation of VC include not only passive calcium deposition but also active osteoid secretion by resident cells (especially vascular smooth muscle cells (VSMCs)) [8]. The adverse microenvironment, including dysregulated mineral metabolism-related hormones (fibroblast growth factor-23 (FGF-23), parathyroid hormone, and vitamin D), divalent ion imbalances, and/or the downregulation of mineralization inhibitors prompts the *trans*-differentiation of VSMCs into osteoblast-like cells with a secretory phenotype [9]. Uremic toxins [10] and epigenetic machineries (microRNAs and methylation abnormalities) [11] also participate in the progressive courses of VC. The complex pathogenesis of VC creates an unformidable challenge for identifying effective therapeutics. Despite the uncovering of potential candidates, very few enter clinical trials and none is recommended by international guidelines [12]. Therapeutic nihilism therefore prevails regarding how to treat VC [13].

The advancement in computational biology and high-throughput platform for target/molecule screening greatly facilitates our understanding of disease pathophysiology and novel treatment identification, including VC [14]. Compounds of either synthetic origin or natural molecules have been shown to be a rich reservoir for procuring potential therapeutic candidates [6]. Druggable targets inherent to the pathogenic landscape of VC can also be discovered through similar strategies. Using a transcriptomic approach followed by experimental validation, we previously showed that astaxanthin might beneficially influence VC severity through its action toward superoxide dismutase 2 (SOD2) [15]. Machine learning, a new strategy and expanding research discipline that trains computers to learn from massive data, emerges as a promising tool to help mine patterns or linkages [16]. The application of machine learning to the field of medicine has fueled and accelerated the progress of precision medicine. Existing literature concerning the use of machine learning in VC studies unanimously focuses on image interpretations and cardiovascular risk stratification, but rarely on plausible therapeutic candidate selection [17]. Furthermore, the process of new drug discovery is costly and time consuming with low success rates. We need more economically efficient ways such as existing drug repurposing, which aims at maximizing the potential usage of existing drugs [18]. As the effective clinical treatment for VC remains a largely undercharted field, we aimed to use a deep learning-based strategy to screen and identify existing compounds capable of being repurposed for managing this dreadful disorder.

## 2. Material and Methods

### 2.1. Overall Workflow of Database Integration and Deep Learning

**Figure 1:**
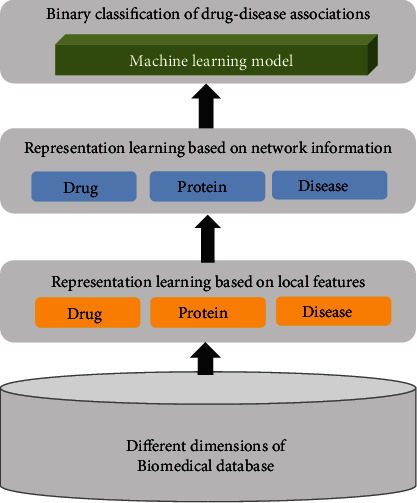
A schematic overview of our workflow.

We used the representation learning strategy, which is aimed at providing a meaningful embedding vector for each node in heterogeneous graphs in order to facilitate the binary classification application of the drug-disease association [19, 20]. A gross overview of our workflow is illustrated in Figure 1. In step 1, we combined the drugome, interactome, and diseasome information from different biomedical database to analyze drug-disease associations. In step 2, we learned a high-level description of the local network architecture and features of the entities using a deep representation learning. This step encoded the content embedding for each node; for instance, the drug might have its own chemical structure for functionality and interactions with its confirmed or predicted target proteins as a subnetwork. In step 3, we learned the global embeddings of the nodes derived from the heterogeneous network using the graph neural network architecture, which was treated as an input into a classification task. This step aggregated the content embeddings from different neighbors and then combined them together by considering different impacts from different nodes. In step 4, we applied a random forest classifier to predict novel associations based on the embedding vectors of the selected drugs and the disease(s) of interest.

### 2.2. Integrating Heterogeneous Biological Information between Drugs and Diseases

**Figure 2:**
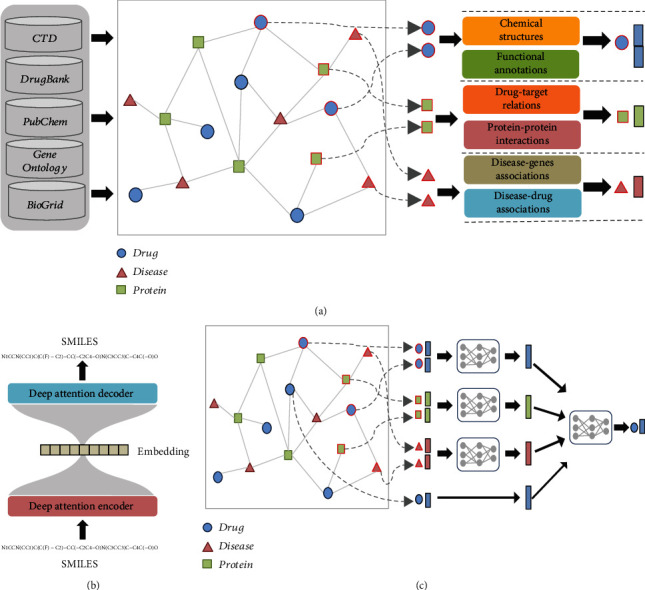
The steps of representation learning in our model. (a) Integrating different dimensions of biomedical database into a heterogeneous network and representation learning with their local content features. (b) The end-to-end transformer for chemical SMILES representation learning. (c) Aggregating the local content features to global features through the heterogeneous network using the graph neural network. CTD: Comparative Toxicogenomic Database; SMILES: Simplified Molecular-Input Line-Entry System.

The fields of “network medicine” and “system biology” are gaining tractions in the recent years. An emerging application of such strategy is to uncover novel associations based on a diverse spectrum of relationships between drugs and diseases [21]. We gathered networks connecting drugs, interactomes, and phenotypes from different biomedical databases and also incorporated heterogeneous information for considering vast arrays of attributes or contents associated with each node in Figure 2(a). The descriptions of each drug were gathered from 2D chemical structures in PubChem, drug-target associations in Comparative Toxicogenomics Database (CTD) [22], DrugBank databases [23], and functional annotations in Gene Ontology (GO). The CTD database provides the disease phenotype-genotype associations and disease phenotype-drug associations. We curated 9,726 drugs, 25,482 target proteins, and 7,238 diseases, while interactions among proteins of human origin were extracted from BioGrid containing 15,352 unique proteins and 281,862 interactions. We used the enriched GO annotations, which were statistically significant GO terms with a *p* value < 0.01 for compounds recorded in the CTD database.

### 2.3. Structural Characteristic Embeddings of Chemical Drug Using the End-to-End Transformer

Cheminformatics is aimed at integrating chemistry with information science techniques based on interactions, structural characteristics, and functional properties [24]. We extracted the Canonical Simplified Molecular-Input Line-Entry System (SMILES) format of the drugs from the PubChem database. The structure of the end-to-end transformer deep neural network is shown in Figure 2(b). We applied the pretrained end-to-end transformer to 83,000,000 SMILES collected from PubChem to obtain the structural characteristic embedding vectors of each drug using the end-to-end transformer deep neural network based on their frequency and sequential order of each SMILES character. An encoder layer with self-attention operation mapped the SMILES sequence into the latent space based on its relationship with other characters [25]. A decoder layer had similar structures to encoder layers, and the output of the final decoder layer was the same as the input sequence (Figure 2(b)). The output of the final encoder layer provided the structural characteristic embeddings of a chemical compound, representing the abstract features for describing the structural characteristics of a chemical drug. We further applied the *t*-distributed stochastic neighbor embedding (t-SNE) algorithm to visualize complex high-dimensional content embedding vectors in a two-dimensional space.

### 2.4. Functional Characteristic Embeddings of Chemical Compounds

To study functional properties, we gathered drug-GO term relationships as bipartite graphs and harnessed *node2vec*, an algorithmic framework for learning the functional characteristics embeddings of the chemical drug in bipartite networks [26]. We first used a biased random walk algorithm to sample sequential paths with different lengths to capture indirect relationships, or high-order dependencies in the bipartite graph. We treated the sequential paths as sentences in natural language and applied the Skip-gram model to generate low-dimensional and real vector space representing the functional characteristics embeddings of each chemical compound. Importantly, compounds with similar functional properties could be mapped closer to each other on the latent space.

### 2.5. Representative Learning of Nodes in Heterogeneous Network Using Graph Neural Networks

Besides the chemical structural and functional characteristic embedding vectors of drugs, the embedding vectors of the diseases and proteins are also learned from disease-gene and drug-target protein interaction networks using the *node2vec* algorithm. After obtaining initial embedding vectors, we summarized a graph-level latent vector considering the heterogeneous structural information via graph deep neural network architectures (GNN) as in Figure 2(c) [27]. We analyzed the latent vectors with the geometric relationships which reflected the topological relationships between nodes. GNN iteratively updated the initial embedding vectors learned from the features of the nodes by propagating the information from their neighbors based on the attention mechanism [27, 28]. An aggregation function took the current embedding vectors of the neighbors of a node and created comprehensive node embedding that maximized effects from the neighborhoods of nodes with different weights as global embedding vectors.

### 2.6. Random Forest Classifier for Binary Classification between Drugs and Diseases

**Figure 3:**
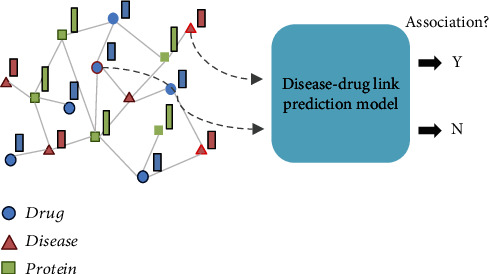
Binary classification of drug-disease associations based on the embedding vectors of the selected compounds and the disease(s) of interest.

We combined the drug and disease global embedding vectors together as the input of the random forest classifier for predicting associations between drugs and diseases (Figure 3). We adopted known drug-disease associations in CTD as the gold standard and applied fivefold cross-validation to evaluate the performances of our constructed model. We randomly split all known drug-disease associations into five equal-sized subsets, combined four subsets as the training set, and treated the other subset as the testing set. We estimated the average of performances ranging from 0.65 to 0.76 using the area under the receiver operating characteristics curve (AUC) value on 5-fold cross-validations. Plant compounds with a probability score predicted by our model higher than 0.5 were selected for testing in experimental models of VC, as detailed below. We also selected one candidate with a low probability score for validation.

### 2.7. Comparing the Embedding Vectors Based on Different Information: The Similarity-Based Strategy

We used a similarity-based strategy to compare the embedding vectors based on different information supported. Similarity-based compound virtual screening using the sparse and binary fingerprint or functional annotation is one of the fundamental methods of preclinical drug discovery. This strategy harnessed computational approaches to predict properties and potential biologic effects of compounds. For comparison purpose, we used the aforementioned dense embedding vectors based on structural (SMILES-only) using end-to-end transformers and chemical functional activities (GO annotations-only) derived from the *node2vec* algorithm. We chose the cosine similarity measurement to formulate the diversity via the angular between different vectors in the embedding, and the score lied within the interval [0, 1], where the value 0 or 1 indicated an extremely dissimilar or extremely similar probability to the reference standards, respectively. Particularly, the diversity mainly provided a representative model with more complement information for the machine learning process [29]. We considered two structural embedding vectors to be significantly similar if the cosine similarity scores were above 0.85 between them, according to prior reports [30].

### 2.8. Testing Compounds for Therapeutic Use against the Predicted Phenotype, VC

We harnessed an experimental model of VC established previously [10, 11, 15] for testing identified compounds. Briefly, a VSMC cell line, A7r5, between 3 and 6 passages was obtained from American Type Culture Collection, plated onto 24- and 6-well plates, and cultured with Dulbecco's modified Eagle's medium (DMEM) supplemented with fetal bovine serum (FBS), streptomycin/penicillin in 5% ambient CO_2_ under 37°C. For the induction of biomineralization, culture media of cells reaching 80% confluence were switched to conditioned ones containing 2.5 to 3 mM inorganic phosphate and adjuvants, resembling the calcification-prone milieu observed in patients with CKD or those of advanced age. Condition media were exchanged every 2 days. After 7 days of treatment, cells would exhibit morphological alterations from being spindle-shaped to assuming a flattened and distended contour, accompanied by prominent extracellular calcium deposition. We qualitatively examined the extent of VC using Alizarin Red staining, with calcifying regions showing reddish areas in nodular aggregates.

For quantitative examination of VC severity, we used the acid elution method; 0.6 N hydrochloric acid was added to the treated and control group cells and sustained overnight followed by phosphate-buffered saline (PBS) wash. A calcium detection kit (ab102505; Abcam, Cambridge, UK) was then applied, in which chromogenic agents were admixed with eluents. We detected absorbance at 575 nm using a spectrophotometric reader, and results were normalized to cell counts obtained after trypsinization by trypan blue counting using a hemocytometer.

In order to test whether selected chemicals exhibited potential for treating VC, we compared calcification quantitation results between the control group, the calcified group, and the calcified group treated with the designated molecules, using Student's *t*-test. Sulforaphane, daidzein, and catechin were obtained from Sigma (SI-S6317, SI-D7802, and SI-C1788; St Louis, MO, USA). For calcium quantitation, experiments were obtained in repeated experiments.

## 3. Results

From the cross-linked database, we collected 6,790 drugs with available SMILES data in PubChem and 11,958 GO terms to analyze the features of compounds. A local embedding vector among nodes learned by an end-to-end transformer network and the *node2vec* algorithm and aggregated the information for global embedding vectors from a heterogeneous network via the graph neural network. A binary classification algorithm of drug-disease associations was conducted for triaging compounds. We focused on natural compounds from plants as the sources of new therapeutic drug candidates, a strategy also adopted by others [31]. For test candidate selection, we referred to the CTD database, in which compounds exhibiting a direct evidence for a drug-disease association were marked as having a potentially “therapeutic role.” For other compounds not listed in the CTD database, we considered them to have unknown drug-disease associations, and these compounds were assigned a probability score predicted by our model.

**Figure 4:**
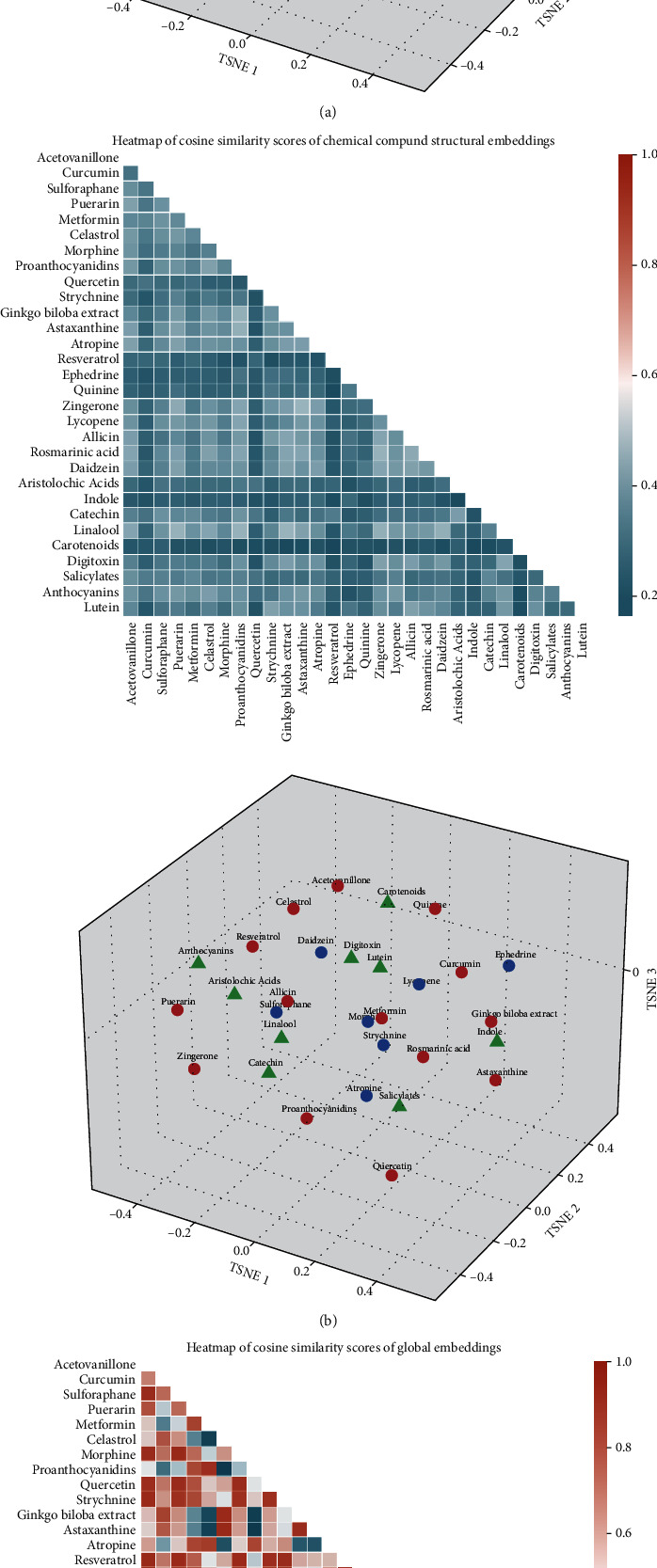
The cosine similarity scores and t-SNE projection of different feature representations among 30 plant-derived compounds based on (a) chemical SMILES embedding vectors, (b) GO functional embedding vectors, and (c) global embedding vectors. Compounds with a higher probability score predicted by our model, and also, direct evidences in CTD and published reports were shown in red circles. This with a higher probability score but without evidence supported were in blue circles, while the rest with a low probability score were represents by green triangles. CTD: Comparative Toxicogenomic Database; GO: Gene Ontology; SMILES: Simplified Molecular-Input Line-Entry System; t-SNE, *t*-distributed stochastic neighbor embedding.

We randomly selected 30 plant-derived compounds after an extensive review of phytomedicinal reports [32], in conjunction with results derived from structure-only embedding vectors and those from functional annotation-only embedding vectors. We examined the similarity scores between any two out of these 30 compounds to see the diversity among different groupings. This was visually displayed by applying t-SNE plots with different feature embedding vectors supported and observing whether compounds belonging to the same category might come close together but different categories remained separated in the t-SNE plots. We found it difficult to differentiate between different categories of compounds in the t-SNE plots based only on chemical structure (SMILES) embedding vectors (Figure 4(a), A) or functional annotations feature embedding vectors (Figure 4(b), A), since the similarity scores were mostly low between any pair of compounds. Some of the plant-derived compounds with cosine similarity of their structural embedding vectors were above 0.85, but similar structural embedding vectors of the drug compounds did not always reflect similar functional bioactivities in general (Figures 4(a), A and 4(b), A). Clusters of potential compounds (red circles, green triangles, and blue circles) tended to mix together without a clear distinction in the 3-dimensional t-SNE plots (Figures 4(a), B and 4(b), B). The first two t-SNE dimensions did not provide a clear separation between clusters, especially when labels were not used for the t-SNE algorithm. It is likely that the biological associations between drugs and diseases are quite complex and that unidimensional groupings based on structure or function-based strategies may not be able to reveal the exact landscape of patterns responsible for the observed drug-disease associations. On the other hand, the cosine similarity scores among our global embedding vectors became more diverse than those using structure or functional vectors (Figure 4(c), A). Ours conferred a greater distinction between potential compounds, presenting as higher similarity scores for the same vector-based categories of compounds but lower scores for different compound categories. Results on a t-SNE plot illustrated the better differentiation ability between 3 groups of compounds (Figure 4(c), B) compared to the traditional approaches (Figures 4(a), B and 4(b), B).

**Figure 5:**
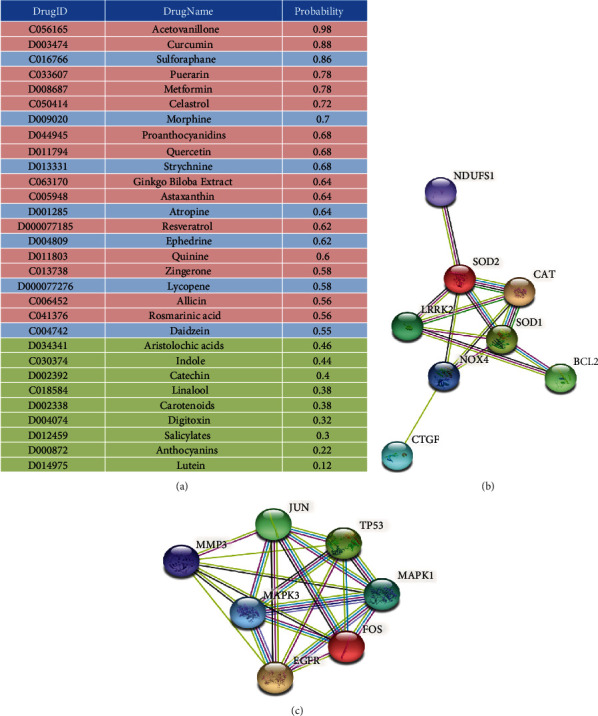
Results of our machine learning-based model including (a) the probability scores of the 30 plant compounds predicted by our model and the interactome analyses of (b) sulforaphane and (c) daidzein regarding relevant expressions in pathogenic molecules involved in VC.

The probability scores of the 30 plant compounds predicted by our classification model are listed in Figure 5(a). Three categories were recognized, including those with possible drug-disease relationship (probability scores > 0.5) and were affirmed in CTD or existing reports (shown in red), those with possible drug-disease relationship but never tested before (shown in blue), and those without drug-disease relationship predicted (score < 0.5) while also without prior evidence (shown in green). Compounds within the red category (Figure 5(a)) have already been validated in the existing literature and were compatible with our prediction results, so we did not choose test candidates from this category. To examine the validity of our model, we purposefully selected three compounds, two from the blue category (possible drug-disease relationship but without prior evidence) and one from the green category (with low probability and without evidence, either) for experimental validation (Figure 5(a)). The former group included sulforaphane (probability score 0.86, the highest ranked one in blue category) and daidzein (probability score 0.54, the lowest ranked one in blue category), and the latter group included catechin (probability score 0.4, in green category) (Figure 5(a)). The selection of sulforaphane and daidzein was based on the presumption that those with the highest and lowest probability scores in the blue category could encompass the entire spectrum of candidates with plausible therapeutic efficacy but without experimental evidence.

Sulforaphane is an antioxidative nutraceutical enriched in cruciferous vegetables [33], and daidzein is a plant-origin isoflavone [34]. Catechin, a green tea constituent [35], serves as a comparator with an estimated neutral effect. None of these molecules has been tested for effects against VC in the existing literature. Functional annotation and interactome analyses showed that sulforaphane (Figure 5(b)) and daidzein (Figure 5(c)) could influence the expressions of various genes that played an important pathogenic role during the course of VC [6].

### 3.1. Comparing Our Approach to Other Machine Learning or Predictive Approaches

We evaluated the performance of our approach by comparing our results with those from the state-of-the-art drug-disease association prediction methods. We adopted the Laplacian regularized sparse subspace learning (LRSSL) benchmark dataset including 3,051 drug-disease associations between 763 FDA-approved drugs and 681 diseases [36]. We constructed three models by combining one of the drug similarities metrics of the chemical substructures (SCMFDD-Chem), protein domains of drug-targeted proteins (SCMFDD-Domain), gene ontology information of drug-targeted proteins (SCMFDD-GO), and disease semantic similarity metrics in SCMFDD method [37]. We adopted *F*1-measure (*F*1) as the primary metric, which was defined as the harmonic mean of recall and precision in highly skewed gold standard benchmark datasets. The results, outlined in Table 1, showed that our approach achieved a *F*1 of 0.724, which was higher than those from the previous studies. These data strongly support the reliability of our model in predicting drug-disease interactions.

Previous studies mainly focused on matrix factorization methods for drug or disease similarity matrix. We also applied the fusion approach introduced by Wang et al. [39] to all drug-related and disease-related similarity matrices to construct two fused drug and disease similarity matrices. Then, we used traditional machine learning classification models such as random forest for drug-disease association prediction. *F*1-measure (*F*1) was used as the primary metric as well. Results are outlined in Table 1, which showed that the representation learning using GNN could capture more semantic meaning of drug and disease than similarity metrics fusion method could do.

### 3.2. Testing the Therapeutic Efficacy of Selected Candidates

**Figure 6:**
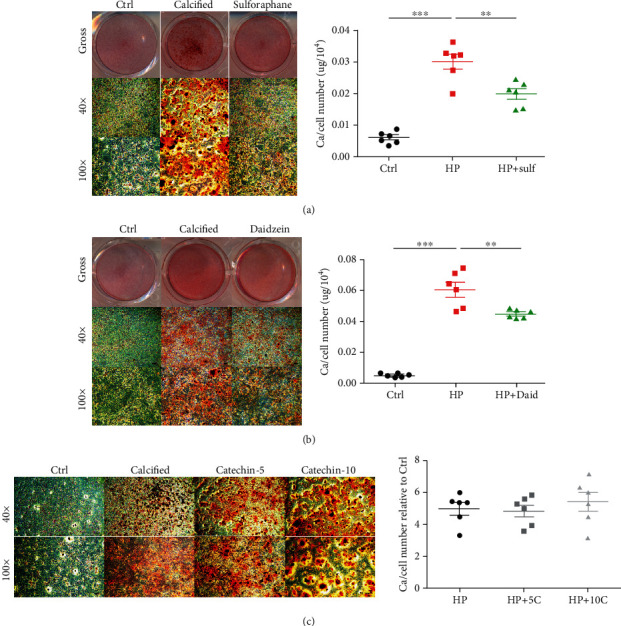
Validating the positively predicted candidates (a) sulforaphane, (b) daidzein, and (c) catechin, a candidate with a neutral effect in an *in vitro* vascular calcification model. Calcification nodules were stained using Alizarin Red staining and imaged without (upper row) and with different magnification levels (mid and lower rows) under microscopic examinations. Comparisons of calcium deposition quantities were done between groups using Student's *t*-tests. ^∗^*p* < 0.05, ^∗∗^*p* < 0.01, ^∗∗∗^*p* < 0.001. C: catechin; Ctrl: control; Daid: daidzein; HP: high phosphate; Sulf: sulforaphane.

Prominent calcification could be observed in VSMCs subjected to conditioned media, both under gross and microscopic examinations (Figures 6(a) and 6(b)). VC *in vitro* manifested as reddish-brown appearing calcification nodules scattered throughout the examined fields (Figures 6(a) and 6(b)). Quantitatively, a 5- to 8-fold increase in the amount of calcium deposited occurred in the calcification group compared to that in the control group (Figures 6(a)–6(c)).

After adding 0.1 *μ*M sulforaphane, significantly fewer calcification nodules were found under gross and microscopic examination, with around 40% to 50% reduction in the eluted calcium amount (Figure 6(a)). Similarly, 10 *μ*M daidzein prominently attenuated the extent of calcification in treated VSMCs, with 30% to 40% lower calcium deposition compared to the nontreated group (Figure 6(b)). However, when adding catechin to calcified VSMCs ranging from 5 to 10 *μ*M, there was no significant change in the extent of calcification nodule development (Figure 6(c)). Calcium quantitation results also revealed insignificant differences between catechin-treated and nontreated groups (Figure 6(c)).

## 4. Discussion

In the current study, we combined a deep learning approach and experimental studies to unravel potential therapeutic compounds capable for treating VC. We harnessed multiple data sources, including CTD, DrugBank, PubChem, OMIM, and BioGrid, for retrieving chemical structures, genomic/protein/functions/disease relationships and their mutual interactions to expand the biologic landscape being integrated. CTD, as the anchor source for platform integration, suited our aim ideally since CTD is a prestigious literature-based database containing an extensive list of chemicals, genomics, phenomics, diseases, and exposomic information [22]. Through representation learning from deep neural network architectures, we curated a machine learning model for scoring to each compound regarding their probability of having a therapeutic relationship with VC. We subsequently tested candidate compounds with a plausible effect and those with a low probability in a well-established *in vitro* VC model. We were able to show that the predicted compounds worked well, while low probability compound exhibited a neutral effect. Our findings can be inspiring both from the methodological comprehensiveness and the validity of predicted compounds and serve as an example for the utility of deep learning in identifying promising agents to treat vasculopathy. The representation learning and deep learning model can be a quick and inexpensive computational screening tool to assist in the early drug discovery process.

Previous studies integrated multiple sources of drugs, genomic, and disease phenotype features such as drug target proteins, side effects, chemical fingerprints and omics data, and the network measurements of the interaction network for drug-disease interaction prediction. Moreover, existing approaches considered the similarity information of drug or disease for investigating common characteristics without considering the topological information between drugs and diseases [36, 37, 40–42]. Therefore, there is ample need for developing a graph-based model to capture the heterogeneous information from the network. Therefore, in this study, we established and validated a representation learning model using GNN for drug-disease association prediction in order to achieve drug repurposing.

Effective drug screening and applicability repurposing remains a daunting task for scientists. A “chemogenomic” approach has long been used to predict potential drug compounds that exhibit efficacy against certain diseases/phenotypes based on chemical-protein interactions and structure-activity relationships [43]. Since the inception of high-throughput screening, the popular approach evolves to studying diseases/phenotypes using mass profiling of cellular/tissue/biological fluid samples from diseased and nondiseased populations, followed by data alignment and comparison, screening for differentially expressed genes/mRNAs/noncoding RNAs/proteins/metabolites, effect size ranking, with or without further input from other adjunct data sources. Results from this newer approach assist greatly in uncovering influential signaling pathways or mediators that count for the disease/phenotype of interest [44]. This strategy can be credited for its straightforwardness and speed in pathogenesis investigation, and its results expectedly facilitate the choice of candidates targeting the newly identified pathways. However, a vital linkage between the identified pathogenic signaling and the selection of candidate drugs is frequently made arbitrarily, attenuating the probability of uncovering novel treatment molecules. Moreover, the conventional high-throughput screening approach rarely addresses the role of compound structures in mechanistic explanations and forgoes the chance of yielding more drug candidates if researchers put little emphasis on the importance of chemical structures in drug screening [45]. In this study, we planned to reap the benefits from both the chemogenomic approach and the phenotype-based high-throughput screening approach, through creating an arching linkage between the structure-activity-relationship and amassing big data from multiple omic disciplines, accomplished by machine learning. A similar approach has been promoted for antineoplastic drug and more recently in antiviral drug virtual screening [46]. In addition, the process of drug repurposing for VC can be further sharpened by combining therapeutic responses with molecular markers for classifying the severity of VC [47, 48].

The strength of our strategy lies in the application of deep learning to selecting therapeutic compounds, an approach that contrasts sharply with other studies harnessing machine learning and big data in the field of VC. Indeed, a recent state-of-the-art review about machine learning and artificial intelligence applicability in cardiovascular calcification addresses mostly calcification imaging and quantification, early diagnosis, and outcome prediction [49]. Regarding new therapeutic candidate screening, researchers rarely utilized the machine learning-based approach. Luechtefeld and colleagues used an expanded database of candidate molecules in combination with toxicological features extracted from sources of regulatory authorities to predict whether candidates exhibit health hazards [50]. They derived fair results based on such approach. However, VC-targeting therapeutic compound screening using a network medicine and machine learning-based approach had not been attempted before. From this perspective, we believe that our findings have their merits and shed light on future VC treatments.

The efficacy of three compounds predicted by our algorithm was validated in this study. Sulforaphane is a metabolite originating from the myrosinase-assisted hydrolysis of glucoraphanin, which is predominantly found in broccoli cultivars and rapidly absorbed into the body [51]. Sulforaphane has been shown to arrest cell cycles through modulating cyclin expressions, inducing cancer cell apoptosis through altering redox balance and activating caspases, inhibiting histone deacetylase, and inducing autophagy [52]. Sulforaphane also activates nuclear factor-erythroid 2-related factor 2 (Nrf2) and indirectly suppresses nuclear factor-*κ*B (NF-*κ*B) activity, exhibiting a salutary effect in CKD, an important VC risk factor [53]. Prior studies showed that sulforaphane reduced VSMC oxidative stress and attenuated VSMC proliferation as well as phenotype switching [54]. Daidzein, a soybean-derived isoflavone and phytoestrogen, has been known to possess estrogenic effect through its bacterial metabolite equol, anti-inflammatory effect, and antiosteoporotic efficacy [55]. Daidzein, along with other phytoestrogens like genistein, was shown to ameliorate DNA damages in VSMCs and decrease neointima proliferation [56, 57], but none evaluated its effect on VSMC calcification. We were able to demonstrate for the first time that sulforaphane and daidzein could ameliorate VC in addition to VSMC proliferation/migration inhibition, which has not been reported before. These findings support the utility of our cross-platform algorithm. On the other hand, catechins, the main antioxidants contained in green tea, exhibit variable degrees of reactive oxygen species neutralization and are touted to have benefits against cancer and neurodegenerative disorders [58]. Our algorithm successfully identified this class of compounds as having a lower probability of working against VC, supporting the differentiation ability of our algorithm.

According to experiences from synthetic biology, especially structure-based predictions based on machine learning techniques similar to ours, synthetic bioactive compounds may not necessarily exhibit the same biologic effects (therapeutic or toxicity ones) as their parent compounds [59]. Moreover, the components of incorporated database and network references in the prediction models also influence the applicability of findings across different compounds. Nonetheless, we have integrated a wide array of biomedical database, containing natural neutraceuticals/pharmaceuticals and synthetic bioactive compounds as well. We believe that results from our machine learning-based prediction model can be applicable to both natural and synthetic compounds. In addition, among the three natural compounds tested in this study, sulforaphane has been shown to exhibit a fair oral bioavailability, while daidzein, a flavonoid with polyphenol structure and catechin, both have poor oral bioavailability and possibly suboptimal absorption [60–62]. Theoretically, the oral administration of these compounds may suffer from inadequate therapeutic efficacy partially attributable to these pharmacokinetic disadvantages. However, newer techniques have emerged to facilitate the absorption and distribution of these bioactive compounds, such as liposomal formulation and other nanocarrier-loaded formulation. Therefore, the therapeutic potential of compounds identified in this study remains promising in the future.

Our study has its strengths and limitations. The integration of multiple database and dimensions for virtual drug screening greatly enhanced our ability to detect potentially useful therapeutic compounds for VC. The verification of such effects in experimental models supported the credibility of our algorithm. However, several remaining issues needed further adjudication. First, we did not verify the therapeutic responses of these compounds using *in vivo* models, and there is a possibility that these compounds may not survive *in vivo* tests. Second, VC is a heterogeneous disease entity, and it is plausible that an individual compound may work against VC of a certain pathogenic subtype only but not others. Finally, our algorithm was not expected to be fixed, since the inclusion of more dimensions in data integration or other data sources might sharpen the prediction ability and derive different sets of prediction. More are still needed in order to pursue clinically meaningful treatment for VC.

## 5. Conclusion

In conclusion, we established a prediction algorithm for identifying novel therapeutic compounds against VC, by integrating megadatabase including CTD, DrugBank, PubChem, OMIM, and BioGrid, incorporating chemogenomic components and multiple omic data sources. Several potential candidates were predicted as being efficacious based on scoring thresholds. We validated three out of the predicted compounds, 2 with a positive effect and 1 with a neutral effect, and affirmed our prediction results in experimental studies. We believe that our approach can be credible and facilitate the subsequent selection of optimal therapeutic compounds for VC, a dreadful vascular complication with gender differences and complex pathophysiology [63, 64].

## Figures and Tables

**Table 1:** The performance comparison with the state-of-the-art drug-disease association prediction methods.

Method tested	*F*1
LRSSL [36]	0.202
SCMFDD-Chem [37]	0.303
SCMFDD-Domain [37]	0.309
SCMFDD-GO [37]	0.314
Fusion+RF [38]	0.580
Ours	0.724

GO: Gene Ontology; LRSSL: Laplacian regularized sparse subspace learning; RF: random forest; SCMFDD: similarity constrained matrix factorization method for the drug-disease association prediction.

## Data Availability

The raw data for conducting this analysis are available upon reasonable request to the corresponding author.

## Abbreviations

AUC: Area under the receiver operating characteristics curve
CKD: Chronic kidney disease
CTD: Comparative Toxicogenomic Database
DM: Diabetes mellitus
DMEM: Dulbecco's modified Eagle's medium
FBS: Fetal bovine sera
FGF-23: Fibroblast growth factor-23
GNN: Graph deep neural network architectures
GO: Gene Ontology
IS: Indoxyl sulfate
NF-*κ*B: Nuclear factor-*κ*B
Nrf2: Nuclear factor-erythroid 2-related factor 2
PBS: Phosphate-buffered saline
SMILES: Simplified Molecular-Input Line-Entry System
t-SNE: *T*-distributed stochastic neighbor embedding
VC: Vascular calcification
VSMC: Vascular smooth muscle cell.
